# Correlates of Mental Illness among Older Adults Using Community Welfare Centers in South Korea

**DOI:** 10.1093/geroni/igaf122.3740

**Published:** 2025-12-31

**Authors:** Jung eun Yun, Sangyong Cho, Jihee Kang, Sueun Shin, Minjin Kim, Young Woo Kim, Jin-Ah Sim, Dong-Soo Shin

**Affiliations:** Hallym University, Chunceon, Kangwon-do, Republic of Korea; University of Cincinnati, Cincinnat, Ohio, United States; hallym University, Chunceon, Kangwon-do, Republic of Korea; Hallym University, ChunCheon; Cincinnati University, Cincinnati, Ohio, United States; Hallym University, Chunceon, Kangwon-do, Republic of Korea; Hallym University, ChunCheon, Kangwon-do, Republic of Korea; Hallym University, ChunCheon, Kangwon-do, Republic of Korea

## Abstract

**Purpose:**

This study examined differences by the presence of mental illness among community welfare center users in an urban–rural complex city. As South Korea pursues deinstitutionalization for people with mental illness, welfare centers must adapt their roles to meet community needs.

**Methods:**

A cross-sectional survey was conducted with 153 older adults from two community welfare centers. The dependent variable was the presence of mental illness (0 = no, 1 = yes). Independent variables included mental health confidence, age, gender, education, income, living-alone, self-rated health, exercise, diet, marital status, and social capital (trust in neighbors, mutual help). Logistic regression analysis was performed.

**Results:**

The model was significant (adjusted R² = 0.346, p < 0.001). Older age was linked to lower likelihood of mental illness (OR = 0.89, p = 0.004), while women showed higher likelihood than men (OR = 11.52, p = 0.021). Never-married individuals tended toward higher risk (OR = 4.65, p = 0.107). Mutual help was positively associated with mental illness (OR = 8.30, p = 0.005).

**Conclusion:**

Age and gender were key predictors, while marital status and social capital warrant further study. Higher prevalence among women likely reflects their greater representation in welfare centers. The positive link between mutual help and mental illness may reflect supportive networks among users with illness, while those without illness may be less engaged due to negative perceptions of welfare centers. Findings underscore the need to tailor programs for users with mental illness and strengthen welfare centers as hubs for aging-in-place and self-management.

